# Identification of novel recessive gene *xa44*(*t*) conferring resistance to bacterial blight races in rice by QTL linkage analysis using an SNP chip

**DOI:** 10.1007/s00122-018-3187-2

**Published:** 2018-09-17

**Authors:** Suk-Man Kim

**Affiliations:** 1 Strategic Innovation Platform, International Rice Research Institute, Los Baños, Philippines; 2 IRRI-Korea Office, National Institute of Crop Science, Rural Development Administration, Jeollabuk-do, 55365 Republic of Korea

## Abstract

**Key message:**

Using QTL analysis and fine mapping, the novel recessive gene* xa44(t)* conferring resistance to BB was identified and the expression level of the gene was confirmed through qRT-PCR analysis.

**Abstract:**

Bacterial blight (BB) disease caused by *Xanthomonas oryzae* pv. *oryzae* (*Xoo*) is a major factor causing rice yield loss in most rice-cultivating countries, especially in Asia. The deployment of cultivars with resistance to BB is the most effective method to control the disease. However, the evolution of new *Xoo* or pathotypes altered by single-gene-dependent mutations often results in breakdown of resistance. Thus, efforts to identify novel *R*-genes with sustainable BB resistance are urgently needed. In this study, we identified three quantitative trait loci (QTLs) on chromosomes 1, 4, and 11, from an F_2_ population of 493 individuals derived from a cross between IR73571-3B-11-3-K3 and Ilpum using a 7K SNP chip. Of these QTLs, one major QTL, *qBB_11*, on chromosome 11 explained 61.58% of the total phenotypic variance in the population, with an LOD value of 113.59, based on SNPs 11964077 and 11985463. The single major *R*-gene, with recessive gene action, was designated *xa44(t)* and was narrowed down to a 120-kb segment flanked within 28.00 Mbp to 28.12 Mbp. Of nine ORFs present in the target region, two ORFs revealed significantly different expression levels of the candidate genes. These candidate genes (Os11g0690066 and Os11g0690466) are described as “serine/threonine protein kinase domain containing protein” and “hypothetical protein,” respectively. The results will be useful to further understand BB resistance mechanisms and provide new sources of resistance, together with DNA markers for MAS breeding to improve BB resistance in rice.

**Electronic supplementary material:**

The online version of this article (10.1007/s00122-018-3187-2) contains supplementary material, which is available to authorized users.

## Introduction

Bacterial blight (BB) is one of the most devastating diseases in rice (*Oryza sativa* L.) and is caused by *Xanthomonas oryzae* pv. *oryzae* (*Xoo*). The disease has been observed in rice-cultivation areas of Asia, the western coast of Africa, northern Australia, and Latin America, and yield loss due to BB in Asia is commonly reported as 20–30% in moderate conditions or up to 80% in certain environments (Reddy et al. 1979; Mew 1993; Nelson et al. 1994; Srinivasan and Gnanamanickam 2005). To prevent yield loss, the development of resistant varieties is suggested as the most effective method to control the disease without requiring collateral input from the farmer and having no environmental impact (McDowell and Woffenden 2003; Suh et al. 2009; Kim et al. 2015). However, the use of cultivars with a single major BB resistance gene (*R*-gene) results in resistance breakdown by pathogen evolution and variation (Vera Cruz et al. 2000; Korinsak et al. 2009; Kobayashi et al. 2014; Dilla-Ermita et al. 2017). Thus, identification of new *R* sources and the pyramiding of known *R*-genes are important methods to enhance sustainable and durable host resistance in the BB-resistant breeding program. In particular *R*-gene pyramiding is a useful method to maximize the use of known *R*-genes, which appear to durable and broad-spectrum resistance to various races or isolates of a specific pathogen (Kim et al. 2018). This *R*-gene combining approach with three or four genes were reported in various case of rice resistance breeding studies (Singh et al. 2001; Sundaram et al. 2008; Dokku et al. 2013; Suh et al. 2013; Pradhan et al. 2015).

*Xoo* is a rod-shaped gram-negative bacterium that invades rice tissue through wounds, stomata, or hydathodes (Mew 1987). The disease caused by *Xoo* is vascular, leading to a systemic infection that spreads from the leaf tips and margins, eventually releasing a milky ooze that dries into yellow droplets. In seedlings, plants are killed by BB within 2–3 weeks after infestation, exhibiting kresek, which occurs when the leaves dry out and wilt. In contrast, adult plants may survive, but rice yield and quality are diminished. The disease is prevalent in both tropical and temperate climates with warm (25–30 °C), humid, deep water, and rainy climates, which are beneficial for the spread of this disease, but not commonly found in North America (Sharma et al. 2017). In addition, severe winds causing wounds and excessive nitrogen fertilization also represent favorable conditions for the spread of the disease. Adhikari et al. (1995) assessed the diversity of *Xoo* using 308 BB strains from various Asian countries. The cluster analysis revealed 5 distinct genetic clusters from the collection, and seven pathotypes were determined by inoculation of five differential cultivars with the *R*-gene, demonstrating tight correspondence between clusters and national or regional origin (Adhikari et al. 1995). To date, approximately 43 genes conferring resistance to BB have been identified from various rice sources derived from *Oryza sativa*, wild relatives of rice and artificially induced mutants (Busungu et al. 2016; Dilla-Ermita et al. 2017). Approximately 62% of the total genes are dominant, including incomplete dominant genes, and 16 genes act in a recessive manner (*xa5, xa8, xa9, xa13, xa15, xa19, xa20, xa24, xa25, xa26b, xa28, xa31, xa32, xa33, xa34,* and *xa42*) (Chen et al. 2011; Liang et al. 2017; Vikal and Bhatia 2017). Among the reported BB *R*-genes, only nine *R*-genes (*Xa1, Xa3/Xa26, xa5, xa13, Xa10, Xa21, Xa23, xa25,* and *Xa27*) have been characterized and functionally analyzed, revealing multiple mechanisms of *R*-gene-mediated *Xoo* resistance (Song et al. 1995; Iyer and Mccouch 2004; Sun et al. 2004; Gu et al. 2005; Chu et al. 2006; Tian et al. 2014). *Xa4, Xa7, Xa22, Xa30, Xa31, Xa33, xa34, Xa35, Xa39, Xa40, xa42,* and *Xa42* loci were identified by fine mapping, and various ORFs within target regions were nominated as candidate genes explaining the trait. The BB *R*-genes are evenly distributed throughout the 12 rice chromosomes. Of these genes, 8 are clustered on chromosome 11, whereas genes have not been reported on chromosomes 1, 9, and 10. While the use of BB-resistant cultivars is crucial to control the disease, resistance induced by a single major gene is easily reversed by pathogenic variation or evolution. Therefore, combining known *R*-genes has been recommended by many researchers as a promising strategy to reinforce durable resistance to BB, but accumulating multiple *R*-genes is difficult to achieve simply using phenotypic selection in conventional breeding programs. Given advancements in molecular technology, DNA markers associated with *R*-genes are being used for foreground selection or gene pyramiding to improve resistance in elite lines. Linkage or association mapping offers practical help to detect novel *R*-genes based on high-density DNA marker sets. Recently, the introduction of more efficient and cost effective marker systems assaying single nucleotide polymorphism (SNP) markers enables high-resolution genotyping for breeding applications in numerous crop species, including rice (Thomson et al. 2012). This technological progress facilitates association mapping, linkage mapping, and marker-assisted selection (MAS) (McCouch et al. 2010; Tung et al. 2010).

In this study, we report the use of QTL mapping resulting in the identification of a new recessive *R*-gene for BB resistance from IR73571-3B-11-3-K3 (P6). Evaluation of the degree of resistance of tested varieties was first performed using a range of BB isolates. Based on these results, mapping populations were developed to identify the resistant locus associated with BB resistance by QTL analysis using a 7-K SNP marker set. Key candidate genes known to be influential factors in plant–pathogen interactions were identified by PCR-based markers to identify new BB recessive *R*-genes.

## Materials and methods

### Plant materials

A total of 493 F_2_ individuals were produced from a cross between P6 and Ilpum and used for QTL analysis based on genotyping using the SNP chip and phenotyping based on bioassays. The parent P6 is one of eight parents from the *japonica* Multi-parent Advanced Generation Inter-Cross (JMAGIC) population (Bandillo et al. 2013) that exhibits resistance to Korean BB races and tolerance to salt stress. The other parent, Ilpum, is a high-quality *japonica* cultivar (cv.) from Korea that lacks any BB R-genes. Some F_1_ plants were also backcrossed with Ilpum to generate BC_1_F_1_ plants. F_1_ and BC_1_F_1_ plants were selected by PCR using SSR markers, and the BC_1_F_2_ population was produced by self-pollination.

### DNA extraction and PCR

DNA samples of plant materials were prepared at a final concentration of 50 ng/µl following the procedure described by Murray and Thompson (1980) with minor modifications and treated with RNAse I at 37 °C for 1 h for SNP genotyping. The concentrations of DNA samples were assessed using the Nanodrop ND 1000-spectro-photometer (Thermo Fisher Scientific, Inc., Wilmington, NC, USA) for nucleic acid quantification, and the quality of DNA samples was confirmed by visualization on 1.5% agarose gel. The PCR reaction was performed in an AllInOneCycler (BIONEER, Korea) in a total volume of 25 µl with 10 ng genomic DNA, 0.25–5 µM SSR primer, 200 µM dNTP mix, PCR buffer (containing 50 mM KCl, 10 mM TRIS–Cl (pH 8.3), 3 mM MgCl), and 0.5 U of taq polymerase. The PCR profile was performed at an initial denaturation at 94 °C for 8 min, followed by 32 cycles of denaturation at 94 °C for 30 s, annealing at 55 or 60 °C for 30 s, and extension at 72 °C for 30 s, and a final extension at 72 °C for 8 min.

### Phenotyping through bioassay

To evaluate the resistance of the population to BB isolate HB1009 (K3a), the leaf-clipping method (Kauffman et al. 1973) was used at the maximum tillering stage of plants. The inoculation was performed in a paddy field (F_2_ and F_1_) and under green house (BC_1_F_2_) conditions. According to the standard evaluation methods of the Rural Development Administration (RDA), Korea (RDA 2012), the average lesion length due to leaf damage of three leaves was measured 14 days after the inoculation. Plants with a lesion length of < 3 cm were categorized as resistance (R), whereas those with a lesion length of > 5 cm were categorized as susceptibility (S). For cases with lesion lengths within 3–5 cm, the plants were moderate resistance (MR). Both of R and MR were considered as resistant type in this study.

### Genotyping and linkage mapping

The Infinium 7K BeadChip composed of 384 SNP sets customized for the *indica*-*japonica* SNP chip (ID: GS0011862-OPA) was used for genotyping. To construct a linkage map of the tested population, SNPs exhibiting polymorphisms between P6 and Ilpum were selected by the parental survey. Some SNPs were removed because segregation distortion appeared within SNPs, or anchored positions overlapped with each other. Compared with the expected segregation ratio (1:2:1), segregation distortion was identified using the MAP functionality of QTL IciMapping version 4.0 software (Meng et al. 2015). Based on quality standards for SNPs, only appropriate SNPs were selected and used to construct a genetic linkage map of the mapping population.

### QTL mapping

QTL analysis was conducted using conventional interval mapping for additive QTL (IM-ADD) and inclusive composite interval mapping for additive QTL (ICIM-ADD). Significance thresholds were determined using 1000 permutations defined at *P *≤* 0.05*. “By LOD” and “By Input” functional options in the IciMapping program referred to the grouping and ordering of anchored SNPs. Mapping distance was calculated by recombination frequency using the Kosambi mapping function. For QTL mapping, genotypic data from SNPs and phenotypic data from measurement of lesion length of damaged lines were combined to detect QTLs related to resistance to BB.

### Devolvement of markers to narrow down the target region

Additional DNA markers were developed within the target region to perform fine mapping. A total of 125 PCR-based DNA markers (Supplementary Table 1) were tested to identify polymorphisms in parents of the mapping population. Nine DNA markers within the region were then selected from the Rice Annotation Project Database (RAP-DB, http://rapdb.dna.affrc.go.jp/). Markers for detecting InDel polymorphisms were developed within the target region using the DNA polymorphism database (Shen et al. 2004). To design primers for InDel markers, we followed the method of Kim et al. 2015. CAP markers were developed by direct sequencing of the PCR amplicon to identify specific sites for restriction enzyme using NEBcutter 2.0 (Vincze et al. 2003).

### RNA isolation and quantitative real-time PCR

Total RNA was extracted from rice seedlings using the TRIzol reagent (Invitrogen, Carlsbad, CA, USA) according to the manufacturer’s protocols. The amfiRivert cDNA Synthesis Platinum Master Mix (GenDEPOT, Barker, TX, USA) was used for cDNA synthesis according to protocols provided by the manufacturer. Diluted cDAN was analyzed by using Stratagene MX3005P qPCR System (Agilent) and amfiSure qGreen Q-PCR Master Mix (GenDEPOT, Barker, TX, USA) for gene expression. To evaluate transcript levels, the rice *eEF1*-*α* gene was used as an internal control for qRT-PCR data normalization (Yokotani et al. 2013). Each set of experiments was repeated three times, and the ddCT relative quantification method was used to evaluate the quantitative variation. Primers used to amplify the selected genes are listed in Supplementary Table 4.

## Results

### Screening leaf reactions to different BB isolates

BB *R*-donors P6 and P8 were resistant to BB race K3a, and *R*-gene loci detected from both donors were delimited by the same flanking markers noted in our previous GWAS analysis (not published). We then sought to determine whether these donors contained different *R*-genes. To determine the race specificity of *R*-gene(s) from each strain against the BB isolates, the leaf-clipping inoculation method was performed with a total of 24 BB isolates from Korea (Table 1). Of 24 isolates the *R*-donor P6 exhibited resistance (≦ 5 cm) to 20 isolates but was susceptible (> 5 cm) to four isolates, HB3055, 3079, 4024, and HB4044. P8 was resistance to all tested isolates. No differences in *R*-reactions were noted between P8 and 11325, which harbors *Xa40* closely linked to the detected position. Based on the leaf-reaction results, P6 exhibited resistance to greater than 20 isolates, but obvious differences in resistance to HB3055, 3079, 4024, and HB4044 were noted in P8 and 11325. Leaf-reaction results confirmed that Ilpum was susceptible to all tested isolates (Table 1).

**Table 1:** Leaf reactions of four lines to 24 BB isolates 14 days after inoculation. The three lines P6, P8, and 11325 are resistant to BB isolate K3a (HB1009), and Ilpum is used as a control for susceptibility. Average lesion length was obtained after measuring three leaves

Isolate	Lesion length of tested line and cultivars to 24 BB isolates (cm)			
	P6	P8	11325	Ilpum
HB1009	3.0 ± 2.3	1.5 ± 0.8	1.8 ± 0.5	12.0 ± 2.0
HB1013	2.5 ± 0.0	2.0 ± 0.0	1.0 ± 2.2	10.1 ± 3.1
HB1014	0.5 ± 1.7	0.5 ± 0.0	4.0 ± 2.2	7.7 ± 3.1
HB1015	4.0 ± 0.0	2.0 ± 0.7	1.3 ± 2.0	8.0 ± 0.8
HB2010	0.5 ± 0.6	0.5 ± 1.2	1.0 ± 0.1	10.0 ± 4.5
HB2024	5.5 ± 1.0	2.5 ± 2.0	3.7 ± 1.5	12.5 ± 5.2
HB2038	4.3 ± 0.0	3.5 ± 0.2	2.0 ± 2.0	12.3 ± 3.2
HB3011	0.5 ± 2.4	0.7 ± 0.1	5.0 ± 1.8	10.3 ± 3.0
HB3034	4.8 ± 1.2	4.5 ± 1.8	6.5 ± 0.5	12.3 ± 2.0
**HB3055**	**9.0 ± 2.4**	**3.5 ± 1.7**	4.5 ± 0.7	10.3 ± 3.1
**HB3079**	**10.0 ± 1.2**	**2.5 ± 0.7**	3.5 ± 0.7	11.3 ± 5.5
**HB4024**	**20.0 ± 4.2**	**3.3 ± 1.5**	1.0 ± 0.0	^a^
HB4027	0.5 ± 0.3	0.5 ± 0.0	3.5 ± 0.0	6.0 ± 2.6
HB4030	6.3 ± 3.5	3.5 ± 0.7	6.0 ± 2.1	12.5 ± 3.5
HB4032	5.5 ± 2.8	3.5 ± 0.7	4.0 ± 2.8	10.3 ± 3.2
HB4040	6.0 ± 0.0	4.0 ± 1.4	2.5 ± 1.4	10.0 ± 1.0
**HB4044**	**7.0 ± 2.8**	**3.7 ± 0.5**	2.3 ± 2.1	11.0 ± 2.6
HB4052	5.0 ± 3.5	4.8 ± 0.3	2.0 ± 0.5	7.5 ± 0.7
HB4074	7.5 ± 2.4	4.0 ± 0.0	2.0 ± 1.7	12.3 ± 2.5
HB4079	5.0 ± 0.7	5.0 ± 1.4	4.0 ± 0.0	8.5 ± 0.7
HB4084	4.5 ± 4.9	4.0 ± 0.0	2.5 ± 1.7	11.3 ± 5.7
HB4087	6.5 ± 1.4	4.8 ± 2.0	2.3 ± 2.1	6.0 ± 2.8
HB5004	4.0 ± 2.1	4.0 ± 2.4	3.3 ± 0.5	8.5 ± 3.0
HB6142	5.5 ± 2.1	3.5 ± 0.7	3.5 ± 1.4	–

Bold indicates a different reaction among P6, P8, and 11325

^a^Is missing data

### Development of the mapping population and R-gene inheritance

To determine the *R*-gene associated with the resistance of P6, a total of 493 F_2_ individuals were produced from a cross between P6 as the *R*-donor and the *japonica* cultivar (cv.) Ilpum that does not harbor any BB *R*-genes. To narrow down the target region associated with the *R*-gene, 42 BC_1_F_1_ individuals were developed by a backcross. Ten BC_1_F_1_ plants selected from the genotype survey using SSR markers were self-pollinated to generate the BC_1_F_2_ population. A total of 520 BC_1_F_2_ individuals were used for fine mapping of the target region associated with the *R*-gene. Phenotyping by measurement of lesion length was performed using each of the F_2_ individuals. The population had a lesion length ranging from 0.1 to 30 cm 14 days after inoculation (DAI). Based on the standard evaluation method, the plants segregated phenotypically as 127 resistant and 366 susceptible lines, fitting the expected phenotypic ratio of 1:3, with *X*^2^= 0.15 and *P *= 0.69 (*P *> 0.05) (Table 2). For the BC_1_F_2_ population, the segregation ratio also followed the expected segregation ratio for a recessive gene of 1:3 (R:S), with *X*^2^= 1.22 and *P *= 0.26 (*P *> 0.05) (Table 2). These findings confirmed a single recessive resistance gene conferring *Xoo* resistance in P6.

**Table 2:** Genetic analysis of BB-resistant response of F_2_ and BC_1_F_2_ from the cross between P6 and Ilpum against the BB race K3a

Cross (generation)	Number of *R*	Number of *S*	Total number	Segregation ratio	*X* ^2^	*P*
P6/Ilpum (F_2_)	127	366	493	1:3	0.15	0.69
P6/Ilpum (BC_1_F_2_)	115	388	503	1:3	1.22	0.26

### Linkage mapping

**Fig. 1:**
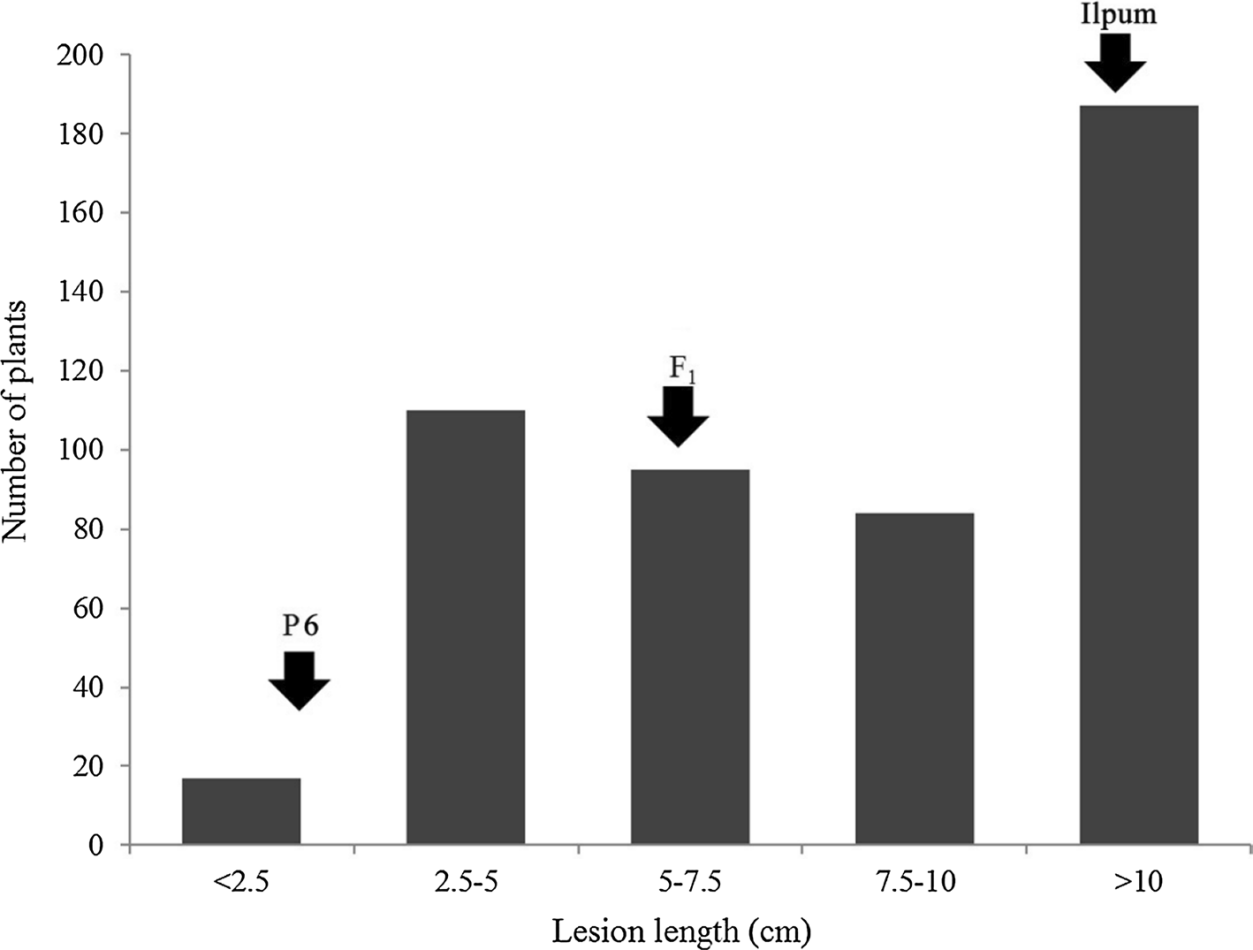
Histogram of lesion length of plants tested using the BB race K3a. Plants were classified as resistant when it had lesion length of less than 5 cm at 14 DAI, and this classification includes both resistant and moderately resistant plants. *R*-donor P6 was resistant to K3a, with lesion lengths of less than 2.5 cm, and Ilpum was susceptible, with lesion lengths greater than 10 cm. F_1_ plants exhibited lesion lengths in the range of 5–7.5 cm

A total of 7098 SNPs were first used to construct a linkage map for the F_2_ mapping population. From the 7K SNP chip analysis, a total of 6658 SNPs were called from the analysis and used for the linkage mapping. Using the parental survey, SNPs exhibiting polymorphic patterns between parents were selected, and heterologous SNPs and deletion and insertion types were removed. From this process, 2983 SNPs were used to construct the linkage map, for an average polymorphism rate of 42.0%. On average, 248 SNPs were anchored on each rice chromosome by QTL IciMapping (Table 3, Fig. 1a). Using linkage map information supported by the software, we detected 1029 redundant SNPs that overlapped the same position on each chromosome with a 0-cM interval. The linkage map was ultimately constructed with a total of 1954 SNPs, with approximately 163 SNPs anchored in each chromosome on average (Table 3).

**Table 3:** Distribution of SNPs on 12 rice chromosomes exhibiting polymorphisms in the parental survey

Chromosome	Tested markers	Polymorphic markers	Polymorphic rate (%)	Selected marker	Selected rate (%)
Chr 1	850	373	43.9	176	20.7
Chr 2	660	273	41.4	104	15.8
Chr 3	683	365	53.4	234	34.3
Chr 4	646	255	39.5	178	27.6
Chr 5	509	219	43.0	180	35.4
Chr 6	551	323	58.6	178	32.3
Chr 7	524	89	17.0	72	13.7
Chr 8	545	219	40.2	148	27.2
Chr 9	455	193	42.4	157	34.5
Chr 10	472	218	46.2	158	33.5
Chr 11	631	284	45.0	184	29.2
Chr 12	572	263	46.0	185	32.3
Total	7098	2983	42.0	1954	27.5

### QTL analysis

**Fig. 2:**
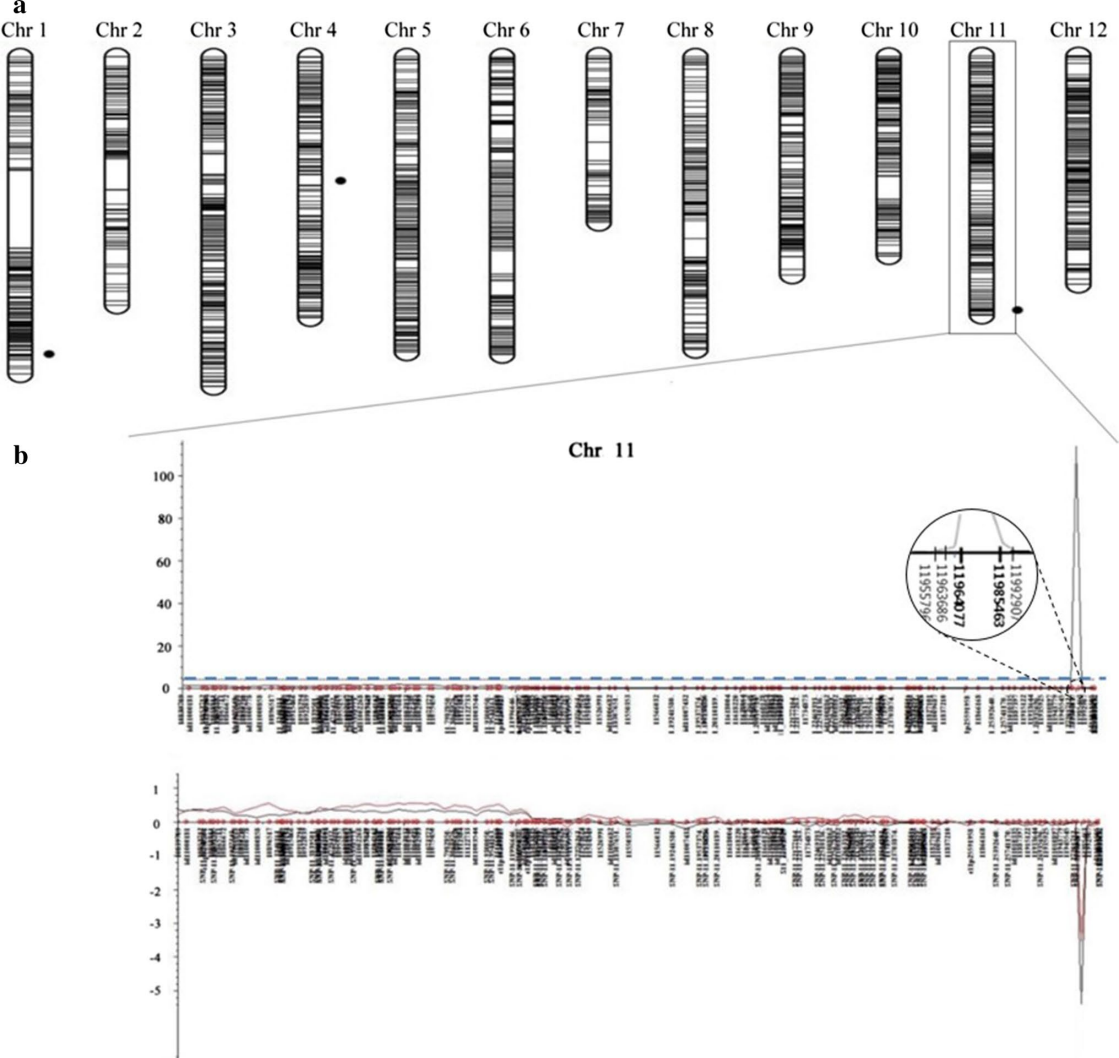
Linkage map and QTL analysis using F_2_ mapping population. **a** Genetic linkage map of the 12 chromosomes based on 1954 SNP markers segregating in the P6/Ilpum 493 F_2_ plants. Black dots beside chromosomes 1, 4, and 11 represent the positions of detected QTLs. **b** The LOD profile and additive effect of major QTL analyzed by inclusive composite interval mapping on chromosome 11. The SNP location was positioned in the whole genome. The positive additive effect was derived from the susceptible parent Ilpum, whereas the negative value was related to the resistant parent. The dotted line is LOD threshold calculated by 1000 permutations

The distribution of BB damage of the F_2_ mapping population revealed a wide range of lesion lengths (Fig. 2). Genotypes of the population were analyzed using 1954 SNPs out of 7K SNP chips and were calculated along with the phenotypic data to detect QTLs associated with BB *R*-genes conferring resistance to *Xoo*. Three QTLs were detected on chromosomes 1, 4, and 11 (Table 4). Of these QTLs, QTLs on chromosomes 4 and 11 revealed a negative influence on the alleles derived from the BB-resistant parent P6, whereas the additive effects of *qBB_1* were positive. One QTL, *qBB_1*, with an LOD score of 9.23 was detected within SNP-1.40447508 and SNP-1.40570714 on chromosome 1, explaining 2.97% of phenotypic variation (*R*^2^) in ICIM analysis. In addition, *qBB_4*, with an LOD of 7.63, was detected within flanking markers (4412887 and 4439600) on chromosome 4 with an *R*^2^ of 2.67. As a major QTL, *qBB_11* detected on the long arm of chromosome 11 and was directly related to P6 resistance to the BB race K3a. The *R*^2^ explained 61.58% in ICIM analysis, with an LOD of 113.59, and this QTL harbored SNPs 11964077 and 11985463. The detected QTL was also confirmed to exhibit a mixture of an additive effect (− 5.39) and dominant effect (− 3.53).

**Table 4:** Putative QTLs associated with the BB *R*-gene from P6 detected by composite and interval mapping

Analysis	QTLs	Chr	Position (cM)	L marker	R maker	LOD	*R*^2^ (%)	Add	Dom
ICIM	***qBB_11***	11	160	11964077	11985463	113.59	61.58	− 5.39	− 3.53
	*qBB_4*	4	81	4412887	4439600	7.63	2.67	− 1.09	− 0.27
	*qBB_1*	1	186	SNP-1.40447508	SNP-1.40570714	9.23	2.97	1.21	0.36

*ICIM* inclusive composite interval mapping, *R*^2^ percent phenotypic variation explained by the QTL, *Add* additive effect, *Dom* dominant effect

### Identification of target regions

**Fig. 3:**
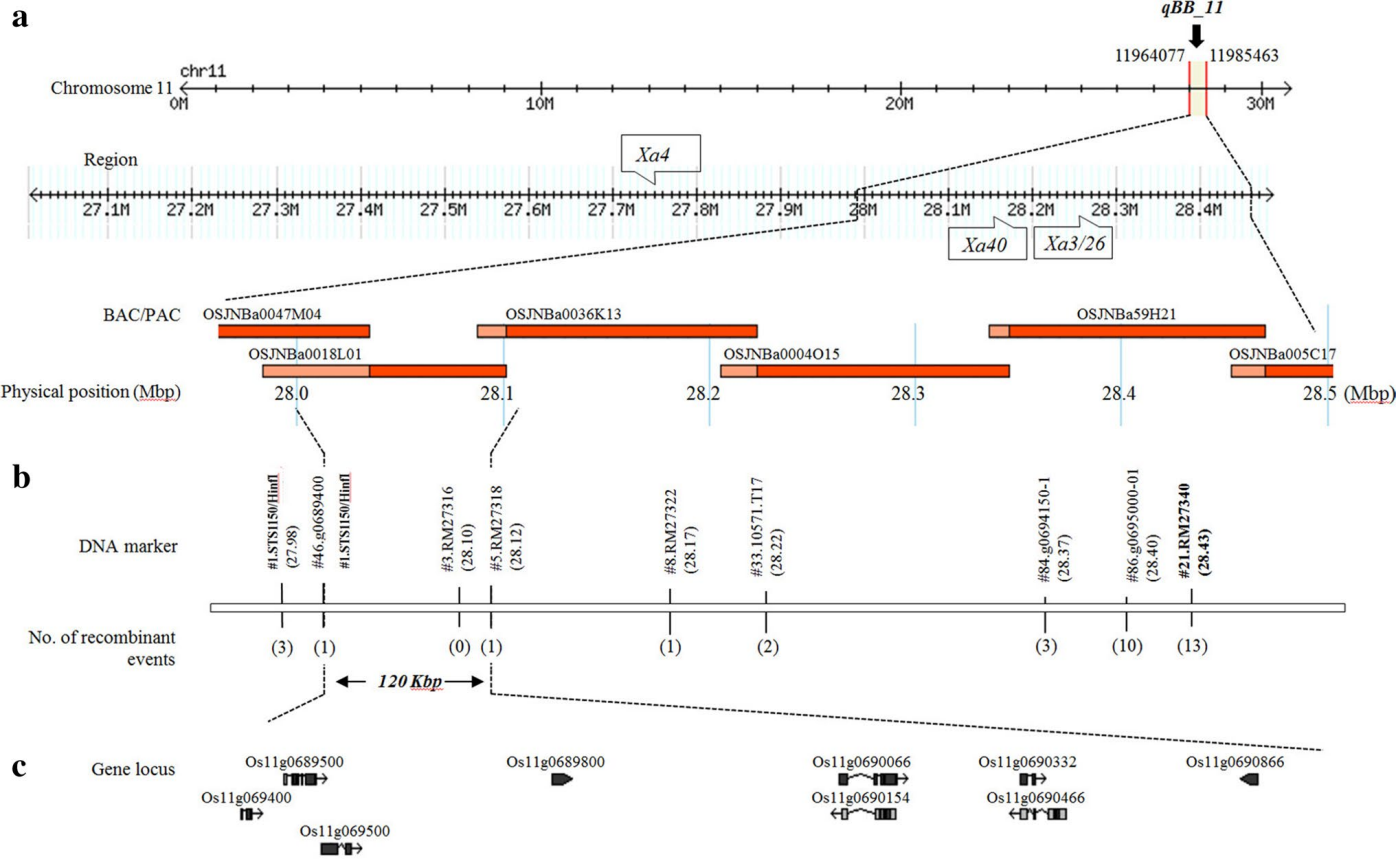
Dissection of *qBB_11* regions. **a** The physical position of anchored marker on the chromosome 11 present based on the Nipponbare genome. Three BB R-genes were clustered near the target region in which loci *Xa40* and *Xa3/26* were included. **b** The target region flanked by the STS marker #1.STS1150/HinfI and SSR marker #21.RM27340 from the QTL analysis. The number in brackets indicates the number of recombinant events detected among P6 × Ilpum BC_1_F_2_ individuals. **c** The loci of ORFs listed in the target region that resulted from the fine mapping based on RAP-DB (IRGSP-1.0). Nine candidates for the BB *R*-*gene* were included in approximately 120-Kbp of the target region

Additional DNA markers were developed to dissect the interval containing QTL *qBB_11*, which is defined by flanking markers 11964077 and 11985463 (Fig. 3a). Of 125 markers newly designed for fine mapping, nine were selected for fine mapping based on the parental survey. Six Nipponbare BAC clones (Load ID: OSJNBa0047M04, OSJNBa001L01, OSJNBa0036K13, OSJNBa0004O15, OSJNBa0059H21, and OSJNBa005C17) were located within in the target region within approximately ~ 490 Kbp, indicating the physical position (Fig. 3a). To delimit the physical location of the *R*-gene, BC_1_F_2_ individuals were used, and sixteen recombinants were finally selected through the analysis of discordance by genotyping and phenotyping. The selected DNA markers were arranged between flanking markers #STS1150/*HinfI*, and #21.RM27340, which are both close to the terminal position (Fig. 3b). According to the number of recombinant events, the target region was further narrowed down toward the left flanking marker #STS1150/*HinfI*. Thus, the *R*-gene is located in the flanking region of the approximately 120-Kbp segment delimited by two DNA markers #46. Os11g0689400 and #5. RM27318 (Fig. 3b). In the case of SSR marker RM27316, no recombinant event was found within the target region (Fig. 3b, Supplementary Table 3). Nine ORFs located in the target region were identified as candidate genes conferring resistance to BB (Supplementary Table 2). Two ORFs, Os11g0690066 and Os11g0690332, encoded proteins related to kinase domain, and the remaining seven ORFs encoded hypothetical protein and non-protein coding transcript, separately.

### Expression analysis of candidate genes in the target region

**Fig. 4:**
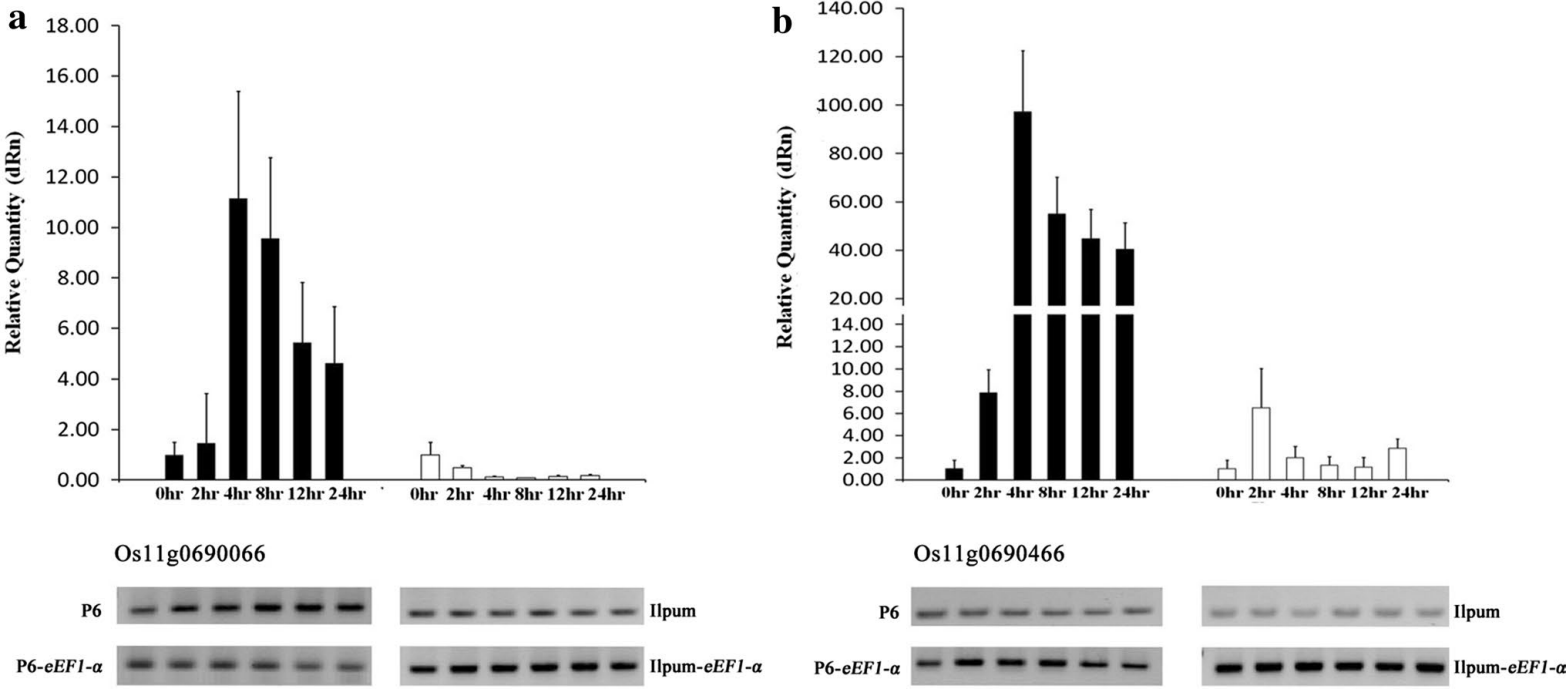
qRT-PCR analysis for gene expression pattern of P6 and Ilpum after BB inoculation. **a** mRNA expression levels of Os11g0690066 at six time-points (0, 2, 4, 8, 12, and 24) after inoculations for both P6 and Ilpum rice varieties, respectively. **b** mRNA expression levels of Os11g0690466 at six time-points (0, 2, 4, 8, 12, and 24) after inoculations for both P6 and Ilpum rice varieties, respectively

An additional primer set was designed to analyze expression levels of candidate genes based on genetic sequence using RAP-DB (Supplementary Table 4). Of nine candidate genes two genes, Os11g0690066 and Os11g0690466, exhibited up-regulation of expression levels in P6 were significantly higher than that in Ilpum (Fig. 4). The expression level of Os11g0690066 in P6 at 4 h after inoculation was observed as significantly up-regulated expression at a level of 11 times (3.48 of log2 fold changes) of the concentration at 0 h (Fig. 4a). In particular the expression level of Os110690466 was considerable increase at 2 h to soar at 4 h and maintained as it declined until 24 h (Fig. 4b). While remaining ORFs revealed no significant difference between both lines (data not shown).

## Discussion

BB is one of the main rice production constraints in many rice-growing areas. Currently, the development and deployment of resistant cultivars carrying major *R*-gene(s) represent the most effective method to control this rice disease. However, the high degree of pathogenic variation in *Xoo* often causes the breakdown of resistance to new virulent BB strains. Therefore, diversifying the germplasm is urgently needed through the identification of resistance genes from wild species and pyramiding two or more effective resistance genes in developing rice cultivars with sustainable BB resistance to *Xoo*.

In this study, we developed an F_2_ population from a cross between the BB *R*-donor P6 and the susceptible *japonica* cv. Ilpum to detect the position of the *R*-gene conferring resistance to the BB race K3a by QTL analysis. To narrow down the detected target region, fine mapping was performed for the BC_1_F_2_ population using additional DNA markers. Based on the QTL analysis, one major QTL *qBB_11* was identified at the end of chromosome 11, exhibiting a resistant response to the tested BB isolate. As a major single recessive *R*-gene, this gene has a high LOD value of 113.59, explaining 61.58% of the phenotypic variation. In previous studies, approximately 43 BB *R*-genes have been reported in rice cultivars, their wild relatives and mutant populations (Busungu et al. 2016). Although recessive resistance is relatively uncommon in plant bacterial systems, the ratio of recessive genes is approximately 38% in nature among all BB *R*-genes, and *R*-genes are distributed on various chromosomes, such as chromosome 1 (*xa34*), chromosome 2 (*xa24*), chromosome 3 (*xa42*), chromosome 4 (*xa31*), chromosome 5 (*xa5*), chromosome 6 (*xa33*), chromosome 7 (*xa8*), chromosome 8 (*xa13*), chromosome 11 (*xa9*), and chromosome 12 (*xa25*) (Korinsak et al. 2009; Liu et al. 2011; Liang et al. 2017). In addition, three recessive *R*-genes *xa15*, *xa19*, and *xa20* were induced in mutant lines (Vikal and Bhatia 2017), and two *R*-genes *xa26* and *xa28* were identified by genetic analysis (Lee et al. 2003). To date, only nine *R*-genes have been isolated and cloned, including *Xa1, Xa3/Xa26, xa5, Xa10, xa13, Xa21, Xa23, xa25*, and *Xa27*, and five types of proteins are encoded by these genes (Gu et al. 2004; Iyer and Mccouch 2004; Sun et al. 2004; Liu et al. 2011; Tian et al. 2014; Wang et al. 2015). Among these genes, three recessive *R*-genes have been characterized. The recessive gene *xa5* encodes the gamma subunit of transcription factor IIA, and recessive gene *xa13* encodes a novel plasma membrane protein that originated from the *aus* genotype (Iyer and Mccouch 2004; Chu et al. 2006; Iyer-Pascuzzi and McCouch 2007). The recessive gene *xa25* derived from *sativa* spp. *indica* encodes a nodulin MtN3 family protein essential for reproductive development and rice–*Xoo* interaction (Liu et al. 2011). Six recessive *R*-genes (*xa8, xa24, xa31, xa33, xa34*, and *xa42*) were identified by fine genetic mapping.

In this study, we identified a new recessive BB *R*-gene designated *xa44(t)* and detected the locus on chromosome 11 by fine mapping. In addition, the locus of the *R*-gene detected in this study contains a closely linked cluster of at least three *R*-genes that were identified in a nearby region as *Xa3/Xa26, Xa4* and *Xa40*. In particular, *Xa3* and *Xa40* were included within the target region (Fig. 3a). However, the *R*-gene *xa44(t)* recessively segregated to the BB isolate comparing adjoining dominant *R*-genes *Xa3* and *Xa40*, and *Xa*3 was particularly susceptible to the BB race K3a (Kim et al. 2015). Thus, bioassays were performed to confirm BB isolate specificity with another *R*-gene, *Xa40*, using 24 isolates in Korea (Table 1). The result showed that *xa44(t)* and the other *R*-genes exhibited different *R*-reactions to four of the tested isolates. As a major QTL, *qBB_11* was eventually identified by linkage analysis, and six Nipponbare BAC clones (OSJNBa0047M04, OSJNBa0018L01, OSJNBa0036K13, OSJNBa0004O15, OSJNBa0059H21, and OSJNBa005C17) were included in the detected target region of approximately 490-Kbp. To further narrow down the target region flanked by SNPs, additional PCR-based markers were developed within the target region and selected by the parent survey. Through fine mapping using 520 BC_1_F_2_ individuals, the *R*-gene *xa44(t)* was delimited to an approximately 120-Kbp segment flanked by DNA markers # 46.g0689400 and RM 27318, and nine putative ORFs involved with the *R*-gene were identified based on the current gene annotation (Fig. 3b, c; Supplementary Table 2). To confirm the candidate gene *xa44(t)*, qRT-PCR was performed using new primer set designed based on the exons of candidates (Supplementary Table 4). Expression levels of two genes Os11g0690066 (Os11g0690154) and Os11g069466 were significantly higher than that of Ilpum (Fig. 4). Os11g0690066 encoded a “serine/threonine protein kinase domain containing protein,” which is known as an influential factor in plant–pathogen interactions (Cao et al. 2011; Lee and Kim 2015). The protein serine/threonine kinase encoded by the candidate has been reported in several crops on disease resistance. BB *R*-gene *Xa21* in rice (Liu et al. 2002), barley stem rust *R*-gene *Rpg5* (Brueggeman et al. 2008), tomato bacterial speck disease *R*-gene *Pto* (Martin et al. 1993), wheat stripe rust *R*-gene *Yr36* (Fu et al. 2009) are related to the protein. Further Os11g0690466 encoding a hypothetical protein was shown to have a much higher expression level in P6 than that of Os11g0690066. However, the function of Os11g0690466 is difficult to define due to lack of identity to protein sequences with annotated biochemical function. In addition, we are also considering possibility that two genes can be actually a single gene due to false annotation like case of *Bph18* (Ji et al. 2016). Given the results of the fine mapping and qRT-PCR, it is highly likely that the two ORFs are related to BB resistance of *xa44(t).*

In this study, we identified a new BB recessive *R*-gene designated *xa44(t)* by QTL analysis and fine mapping using various molecular markers sets. PCR-based DNA markers tightly linked to the *R*-gene were developed for MAS, and key ORFs were suggested as likely candidates for *xa44(t)*. These will be especially useful for BB breeding programs as the *R*-gene exhibits resistance to the four main BB races, K1, K2, K3, and K3a, which represent approximately 95% of races in Korea, conferring broad-spectrum resistance to *Xoo* in Korea. The results provide further useful information to understand BB resistance mechanisms and provide DNA markers for MAS breeding to improve BB resistance in rice. Further studies to characterize *xa44(t)* gene structure, protein sequence, and biochemical function will be required.

### Author Contribution statement

SMK carried out all researches regarding the study and drafted the manuscript.

## Electronic supplementary material

Below is the link to the electronic supplementary material.

Supplementary material 1 (PDF 318 kb)
