# Supplementary material for: Identification of novel recessive gene xa44(t) conferring resistance to bacterial blight races in rice by QTL linkage analysis using an SNP chip
Source: Theor Appl Genet. 2018 Sep 17;131(12):2733–43. doi: 10.1007/s00122-018-3187-2 (PMC6244528; doi:10.1007/s00122-018-3187-2)
Supplement: Supplementary file 1 — Supplementary material 1 (PDF 318 kb) [file 122_2018_3187_MOESM1_ESM.pdf]

Supplementary Table 1. List of used markers

#	Marker	F	R	size
1	STS1150/*Hinf*I	GTGAGCTCCCCGCTTCAT	GTAAGGGGAAGAAGGCAACA	**283**
2	RM27310	TTACCAACCGGGACTAAAGATCG	CAATTCATAACGTCGGTCCTTCC	**498**
3	RM27316	GATCGATCTTTAGTCCCGGTTGG	AACACAATTCACCGGTCCTTAGC	**463**
4	RM27317	AGCGAAGCGCATGGTAATGACG	CTGCCAGCATGACCGAAATCTTCC	**360**
5	RM27318	TGAACTGGACATGCCTTTATCC	ATGCGCCTTACTTTGTTGTAGG	**223**
6	RM27319	GAGCGTTTGTAGGAAGTTTCATGG	GCACCACATTGTCAAATCATGG	**248**
7	RM27321	TATGCATGCTTGTGATCTCC	GGATCCACGAATAAAGAAGG	**196**
8	RM27322	AGAGCCCATGTAGCTACGCCTTCG	AATCATGCCGGCTGAAATTGTACC	**266**
9	RM27325	GGAGGGAGGAGAGAAGGATCAGG	CCGGTGTCCAAACCTCTTACTGC	**573**
10	RM27326	ATCAACGAGTACGCAACAGTCC	TCCTGTCTCTTCACATCCTAATCG	**160**
11	RM27327	TCAATCCCGAATCCAAATCCTTCC	GTTGTGACGACGGCGATGACG	**383**
12	RM27328	CAAGCAGGTACTGAGGCGTAGC	TTATGCGGAACTCCTCCTATATCC	**247**
13	RM7228	CTCGTATTGGGTTCTTGTATGG	AGACTGCTGTTCAGGTGTAATCC	**592**
14	MRG6228	TAGCCACCACAGTGAATGTG	GCTCCACAGGAAAGAAAAGC	**202**
15	RM27333	GGACTCCACATTAGCACCAAACTGC	TTGTTTGGGCACAGCTGTTTCTACC	**442**
16	RM27336	AGCAGTGATTCATCGCTCTATCG	GTGATGCTGTGCCGATAATATTCC	**313**
17	MRG6272	GGTTAGATCGGCAACGATTG	CACGTAGCGTCGTAGGAGTG	**193**
18	RM27337	AGCGTCGTAGGAGTGTTTGTAGG	TGTTGATTGGGTGGTTAGAACG	**200**
19	MRG2577	CCGATCCCATTCACAGATTC	CAGTGCCTTGATCGATGTTG	**131**
20	RM27339	GACGCAGTGTGTCTATGACAGG	CTAGTACACGTGGCGTCGTAGG	**170**
21	RM27340	GTGATGCTGTGCCGATAATATTCC	AGCAGTGATTCATCGCTCTATCG	**313**
22	C5-indel9391	CACCCTAAGACAACACGATCAC	ATCGATCGCTATCAACCACACT	**134**
23	C5-indel9392	ACGGCCAGGTTACATCAGTAAG	GAGGATTAAATGCTGGGTGAAT	**122**
24	C5-indel9393	ACCATATGCCTGCACCTAAATC	GGATGGCATGAGGAAGAGTTT	**147**
25	C5-indel9394	AAACCTTCTCCACAGTCTCCAA	TTACTTCTCTCGGGCCAGATT	**98**
26	Os11g45940 (seq)-1	ACATTTCGAAACGGAGGGAG	TTGGAGAGAGGTTTCGCTGA	**998**
27	Os11g45940 (seq)-2	AGCTCTCGTGTCGATCACCT	GGTAGCATGAAAATGGTGCAA	**299**
28	Indel1	TTCCGACAGCCAGAGATCAT	TGCTGTCATTCAACCATTTAGT	**790**
29	Indel2	AGCTCTCGTGTCGATCACCT	GGTAGCATGAAAATGGTGCAA	**299**
30	K34	ACCTTGGCTCCTGACTGTTG	CCAGGCAGGCTGTTGGTTCA	**671**
31	K27	GGAGAAACGGTTCAGGATGC	TGTCTGGGTGTGTGTGAGTG	**1142**
32	10571.T14	TGTTGGAGGATTGGCAAGGAA	TTCGTTGCGGCGTTGTTAATC	**650**
33	10571.T17	GCAGGCCAAGAACTTGGAGGT	ATCCTTGGCAGCAATTGGACA	**750**
34	Os11ssr0197500	TCCCACGTGAACATCACACT	CTCGTCGCTAATGCCATCC	**244**
35	Os11ssr0197700	GGCTAGCTCTTCCACTCAGG	ACGATCGAACGAAGACAAGC	**242**
36	Os11ssr0197700	GGCTAGCTCTTCCACTCAGG	ACGATCGAACGAAGACAAGC	**244**
37	Os11ssr0197800	GTCATCAATCCATGCCACTG	CCTCTCGGTTGCGTATTTGT	**164**
38	Os11ssr0197800	GATGCGTTTGGCTAGTGGTT	CCTCTCGGTTGCGTATTTGT	**201**
39	RM27299	CAACCGAGAGGGTGAAGGAAGC	CGATTCCACAGCTGCCAACG	**178**
40	Os11g0688832_P6_01	CCTCAGGCTGCTCAAGTACC	CAGCTCGCTGATATTCCACA	**262**
41	Os11g0688832_P6_02	GATTGCGAGTGTTGGACCTT	CAGCTCGCTGATATTCCACA	**341**

42	Os11g0688832_P6_03	TCCCCCTATTGGCATATTCA	ATGATGGGCTCTCTTTGTGG	**204**
43	Os11g0689100_p6_01	GATGCCAAATGCTCCTTCAT	GACTCGGCAGTGTTCTCCTC	**596**
44	Os11g0689100_p6_02	TCATGCTGTTGGTTCCACAT	AGTTGGCTTTGTTTGGGTTG	**257**
45	Os11g0689100_p6_03	CGAAGCAAGATCAGGACACA	ACTCTTGTCCGGAAAGCTGA	**155**
46	Os11g0689400_p6_01	GGCTTCTTAAACCCCACTCC	GGGTAGGGGCCAAAACTAAC	**190**
47	Os11g0689400_p6_02	ATTGCCAAGACAGGATCAGG	GTGAAGCAAGCTCTCGGGTA	**133**
48	Os11g0689000_p6_01	CCTCTCGGTTGCGTATTTGT	GTCATCAATCCATGCCACTG	**164**
49	Os11g0689000_p6_02	CGGGAAGTTTGTGTGGAGAT	TCGCCTCCTCTACCCTTTTT	**141**
50	Os11g0689300_P6_01	ACGCAGTCTTCTCAGCCAAT	GTTGGTCGCAAAGCGATAAT	**269**
51	Os11g0689300_P6_02	TTCTTGGGGAATTGTCTTGG	TGCTAGCAACCTCCAACCTT	**249**
52	Os11g0689300_P6_03	ACGCAGTCTTCTCAGCCAAT	GTTGGTCGCAAAGCGATAAT	**269**
53	Os11g0691100_P6_01	GCGCAACAGATATGGAGTGA	ACAGTCTTCATCCCCACAGG	**153**
54	Os11g0691100_P6_02	TCATGCTGTTGGTTCCACAT	AGTTGGCTTTGTTTGGGTTG	**257**
55	Os11g0691100_P6_03	GAGGAGAACACTGCCGAGTC	ATGTGGAACCAACAGCATGA	**461**
56	RM27320	GTTAACCCAGCTTGGAAAGACAGC	CCAGTACTGTTGACGAAGCTCTTGC	**250**
57	ID55.WA3	TCCGGCAATATTCTTCTTGG	CCACAAGCGCATATAGCAAA	**689**
58	ID55.WA18-5	GTCGCCGTCAAATACTCCAT	AGGCACTCCATGCAGTAAGG	**713**
59	LRR-1 del	CCAACCCAAAGCAAGTGAAT	GAACCCGTGAGGTTTGTGAT	**795**
60	ID55.OsWA123	TTTAGCTAGGGCACCCATTG	TGCTACCACCGTAACATGGA	**625**
61	ID55.LRR(II)-1	CTTTCCTCCGTGTCCTCAAC	TCCAACAAAAGCTGGAAAGG	**864**
62	ID55.05-79	AAATGGCTCCACAATGAAGG	GATGGGCTCACTGACAGGAT	**162**
63	I_Bb11.2782(88)_F_6	GAAGACAACTTTAGTCTCGGTTGAG	AAGCAAAGGTAGCTTGATGTATTTG	**200**
64	Os11g0688000	AAGAGGCCACCAAAAGTGTG	GCCCCTTGACCTCTTTCTTC	**246**
65	Os11g0688200	GCGGGACTCACATGGTACTT	GGTCCTCCTCTTGACCCATA	**217**
66	LOC_Os11g46130	GATTACCAAGAGGGCCACAA	CCTCCAATGCCAACATCTCT	**239**
67	LOC_Os11g46140	TGAAGATCCTTGGAGGGTTG	CTCGACTCTCCGAACCTTTG	**173**
68	LOC_Os11g46150	CAAGCACTAGCAGCCATCAA	AATGGCGTGCATTGATGTTA	**144**
69	Os11g0690066_1	TCCCTCTATTGGCTCGCTTA	CCAAAGACGGACCACTTGTT	**208**
70	Os11g0690066_2	TGTCCCTAGGTTGGTCTTGG	GACATCACCATGAACGATGC	**181**
71	Os11g0690066_3	AGCCAGTCCTCTATCCAGCA	GACATCACCATGAACGATGC	**243**
72	Os11g0689500_1	GGTGGTTCTGCCAAGAACAT	CTCATCAACAGCAGCCACAT	**159**
73	Os11g0689500_2	TGACATGGAAGCTGATGCTC	TCCTTCGATGGCGATAGTTC	**195**
74	Os11g0689500_3	CACAAGGAGTGATGCTGTGG	GTAGGGTCTGGGAGGGGTAA	**224**
75	Os11g0690332_1	TTCTTCTGTGCTGGTGGCTA	GTAGGGTCTGGGAGGGGTAA	**157**
76	Os11g0690332_2	CACAAGGAGTGATGCTGTGG	GTAGGGTCTGGGAGGGGTAA	**224**
77	Os11g0689650_1	GATGGATCGGCTTCACTGAT	CCCTCAACATGGTTTTGCTT	**221**
78	Os11g0689650_2	GCCAAGCAAGAAAGAACAGC	CTCTCAGCCCCAAAATTGAA	**249**
79	Os11g0691800_1	AACGGCTTAACTGGCCCTAT	TTGAACCACCATCGAAGTGA	**241**
80	Os11g0691800_2	TTGGCAGCATGAACTCTTTG	GTATGGGGCCAAACAAGCTA	**360**
81	Os11g0691800_3	GAAAGCTGAGCCCTCACATC	ATGCAGGCTGGTATTGATCC	**382**
82	Os11g0693750_1	AGAGCACAACCCCAAAGCTA	AATGTCCCAATGCTGGTCTC	**382**
83	Os11g0693750_2	TCTCCAAACAACAACCCACA	TTGCGCATGGAATATGAGAA	**319**
84	Os11g0694150_1	AAATTTTTGACGCCGTTGAC	TGCTAAGGATTGTGCTGTCG	**344**

85	Os11g0694150_2	GTGTTTCTTCACCGCCATTT	TCGGTCAATGATGAGGTTGA	**347**
86	Os11g0695000_1	GAGCTGGAAGTTTTGGCAAG	GCTTGAGGTCACAGTGCAAA	**360**
87	Os11g0695000_2	CCTTGTGTGGTCTTCCTCGT	CTTGCCAAAACTTCCAGCTC	**275**
88	Os11g0695000_3	GTTCTACGCATGGTTCAGCA	GCTTGAGGTCACAGTGCAAA	**241**
89	Os11g0689300_L1_1	TGCTGAATTATGTACTTGGGATTC	GCAAGGACTTGCAAACCTGTA	**851**
90	Os11g0689300_L1_2	GCTGAATTATGTACTTGGGATTCAT	GCAAGGACTTGCAAACCTGTA	**849**
91	Os11g0689300_L2_1	GCAGCCGAGTAGGATTTCAG	TCAGCCTGTTATCGCTGATG	**2614**
92	Os11g0689300_L2_2	TTTCAGGGCTCTAGCCTTCC	TCAGCCTGTTATCGCTGATG	**2600**
93	Os11g0689300_L3_1	AATGCAAGGACTTGGACAAAA	CCGGTGAGACCATTATACGG	**1950**
94	Os11g0689300_L3_2	AATGCAAGGACTTGGACAAAA	CGCCGGTGAGACCATTATAC	**1951**
95	g0696400_1	CATGGACTTCCTCACCACCT	GACATTGACGAAGCCAACCT	**277**
96	g0696400_2	CATGGACTTCCTCACCACCT	GTTCTTGAGGAGGACGATGC	**226**
97	g0696400_3	AGAGGAGATCATCCGTGTCG	GTCATGGGCAGCTGGTAGAT	**328**
98	g0696200_1	CAACCATGAAGTTGGTCGTG	TCCGAGAAGCTAATGGTGCT	**240**
99	g0696200_2	GACTTGTGGCCCTCAACCTA	TCCGAGAAGCTAATGGTGCT	**283**
100	g0696200_3	CCTTCATTGTGGAGCCATTT	CTCCAATGGAAGGGTAGCAA	**209**
101	g0696200_4	GCTGCAAGGATGCACATCTA	TTGCCCATTTGCATTTATGA	**187**
102	11g0695900_1	GGGTTTTGCTGCACTGGTAT	TGGCAATGAAGAGCAAACAG	**291**
103	11g0695900_2	ATGATGGTGATGGTGCAGAA	CGGGTTTACCCAATTCAATG	**323**
104	11g0695900_3	GAGGAGGAAGACACCGATGA	CGGGTTTACCCAATTCAATG	**276**
105	11g0695900_4	TTGCGATGGTCATCAGTCAT	GGAGCACAGACCCATCAAAT	**234**
106	11g0695800_1	AAGCTCACCGTGCTTGATCT	TCCCCTTGCAGATGATTTTC	**260**
107	11g0695800_2	GGGAATCTCTTGACGGATCA	TTCCCAAGGGTCACAGGTAG	**329**
108	11g0695800_3	CATGCAGGTTGAACGAGCTA	AGGACGACCTCATGGTGTTC	**273**
109	11g0695800_4	CTGCCTGTCTGGAGAAGTCC	GCCAAAACTTCCAACTCCAA	**257**
110	11g0695800_5	AACGGTGTTGGCAACCTAAG	CAAGGGGCTGAGATCAGAAG	**162**
111	11g0695800_6	ACTCATGGGAAAGGCATCAC	TAGCCATCCTCTGCTCAGGT	**323**
112	11g0695800_7	CACTGGGAAGAGGCCTACAG	TAGCCATCCTCTGCTCAGGT	**257**
113	11g0695800_8	TTCCCAACTGGAAACCAAAG	GACAAGCCTGCTAGCCATTC	**164**
114	11g0695800_9	TTTCCTCATCGTCCTCAACC	GCCGGTTAAACTGGAGATCA	**179**
115	11g0695400_1	CCGTTGTCTTCCCTGTCAAT	CGGTTAGACCGGAGATTCAA	**242**
116	11g0695400_2	CAACATCGATCAAGGCACTG	AGCCTGGAGAGTCTGTTGGA	**224**
117	11g0695400_3	CAATCCACGTGCATCAACTC	ACTGTGTCGGTTTCGGTTTC	**294**
118	11g0695400_4	TGATCTCTGACCACCACGAA	ACTGTGTCGGTTTCGGTTTC	**256**
119	11g0695000_4	GGCAAGGTTTACAAGGGTCA	GCTTGAGGTCACAGTGCAAA	**346**
120	11g0695000_5	TTTGCACTGTGACCTCAAGC	TCGAGCAACATGATCCCATA	**219**
121	11g0695000_6	GAGCTGGAAGTTTTGGCAAG	TTGGGCATGTACTGAAGCAG	**214**
122	11g0695000_7	GTTCTACGCATGGTTCAGCA	GCTTGAGGTCACAGTGCAAA	**241**
123	11g0695000_8	GGCAAGGTTTACAAGGGTCA	TTGGGCATGTACTGAAGCAG	**200**
124	11g0695000_9	TGGTCAGCTCCCAAATTCAT	ACGAGGAAGACCACACAAGG	**301**
125	11g0695000_10	TGATCTCAGCCACATCCAAG	CAGCATTGCCCATCAAAGAT	**338**

Supplementary Table 2. List of putative ORFs in the target region in

Locus	Putative function	Transcript evidence
Os11g0689400	Similar to predicted protein	CT835640
Os11g0689500	Conserved hypothetical protein	AK242805
Os11g0689650	Conserved hypothetical protein	tplb0033p11
Os11g0689800	Non-protein coding transcript	AK119600
**Os11g0690066**	**Serine/threonine protein kinase domain containing protein**	**AF327447**
**Os11g0690154**	**Hypothetical protein.**	**tplb0049o19**
Os11g0690332	Similar to Protein kinase domain containing protein	-
**Os11g0690466**	**Hypothetical protein**	tplb0049o19
Os11g0690866	Non protein coding transcript	tplb0049o19

Supplementary Table 3. Genotype and phenotype of 16 recombinants between #STS1150/*Hinf*I and #21.RM27340

#	Plant No.	Reaction	#STS1150/*HinfI*	46. Os11g0689400_p	#03. RM27316	#05. RM27318	#08.RM27322	#33.10571.T17	#84.Os11g0694150_1	#86.Os11g0695000_2	#21.RM27340
1	15_5	R	Pa	P	P	P	P	H	H	H	H
2	36_12	R	P	P	P	H	H	H	I	H	H
3	6_12	S	I	I	H	H	H	H	H	P	P
4	7_12	S	H	H	H	H	H	H	H	H	P
5	19_13	S	H	H	H	H	H	H	I	P	P
6	20_1	S	P	H	H	H	H	H	H	H	H
7	20_7	S	I	H	H	H	H	H	P	P	P
8	20_12	S	H	H	H	H	H	H	I	P	P
9	20_13	S	H	H	H	H	H	H	H	H	P
10	22_2	S	P	H	H	H	H	H	H	H	H
11	22_9	S	I	I	H	H	H	H	H	P	P
12	28_10	S	P	H	H	H	H	H	H	H	H
13	33_6	S	H	H	H	H	H	H	H	H	P
14	34_10	S	I	I	H	H	H	H	H	P	P
15	34_12	S	H	H	H	H	H	H	H	P	P
16	40_1	S	H	H	H	H	H	H	H	P	P
Recombinant issue			**3**	**1**	**0**	**1**	**1**	**2**	**3**	**10**	**13**

[a] P: P6 allele

I: Ilpum allele

H: Hetero type

Supplementary Table 4. List of primer sequences used for qRT-PCR

Locus	Primer name	TM	Forward	Reverse	Expected size (bp)
Os11g0689400	qR_1	60	CAAACATGGCCTGTTGCACTG	GCCCAACATTGGGTTGTTTTG	132
Os11g0689500	qR_2	60	AGACAAGCCTGCAGACCCTCA	ATCCTGGATCCTGCATTGGTG	104
Os11g0689650	qR_3	60	CACAAGGCAACAGTGCTCCAA	CCGCGTTTTCAGCGATATTTG	108
OS11g0689800[b]	-	-	-	-	-
Os11g0690066	qR_4 [a]	60	GAGGCGGAGAAGATTGTCGTG	CCGACGAGAAGCAAGAGGTTG	125
Os11g0690332	qR_5	60	GCTTCCACGTCTCTTGCAACC	ACGTCAGTGTACACGCGCATC	112
Os11g0690466	qR_6	60	TCGAAGACCAGCATCGGAATC	AAGCGCCCCAATACGTTTGAT	140
OS11g0690866[b]	-	-	-	-	-
House keeping	*eEF1-α*	60	CCCAGCGTGAGAGAGGTATCA	TCTTGATGAAGTCACGGTGACCA	121

[a] qR_4 is designed based on the sequence of Os11g069066 and Os11g0690154
[b] There is no exon region as non protein coding transcript
